# Correction: Wang, Y.; et al. Magnesium Alloy Matching Layer for High-Performance Transducer Applications. *Sensors* 2018, *18*, 4424

**DOI:** 10.3390/s19183888

**Published:** 2019-09-09

**Authors:** Yulei Wang, Jingya Tao, Feifei Guo, Shiyang Li, Xingyi Huang, Jie Dong, Wenwu Cao

**Affiliations:** 1 National Engineering Research Center of Light Alloy Net Forming, School of Materials Science and Engineering, Shanghai Jiao Tong University, Shanghai 200240, China; 2 Department of Instrument Science and Engineering, Shanghai Jiao Tong University, Shanghai 200240, China; 3 Shanghai Key Lab of Electrical Insulation and Thermal Aging, Shanghai Jiao Tong University, Shanghai 200240, China; 4 Department of Mathematics and Materials Research Institute, The Pennsylvania State University, University Park, PA 16802, USA

The authors wish to make the following corrections to this paper [1]:

In the Results and Discussion section of the paper [1], Figure 7 and Figure 8 from another set of simulations using different parameters were mistakenly used, so the correct ones are given below:

The designed 5 MHz transducer showed a center frequency of 4.73 MHz after putting the backing and matching layers with a −6 dB bandwidth of 77.38% (corresponding to the lower and upper −6 dB frequencies of 2.90 MHz and 6.56 MHz). The center frequency and −6 dB bandwidth for the designed 10 MHz transducer were 9.61 MHz and 77%, respectively (corresponding to the lower and upper −6 dB frequencies of 5.91 MHz and 13.31 MHz). These simulation results agreed well with the experimental results.

In addition, the anti-resonance frequency for the fabricated 5 MHz transducer listed in Table 3 of the paper [1] should be 4.87 MHz, instead of 6.0 MHz.

## Figures and Tables

**Figure 7:**
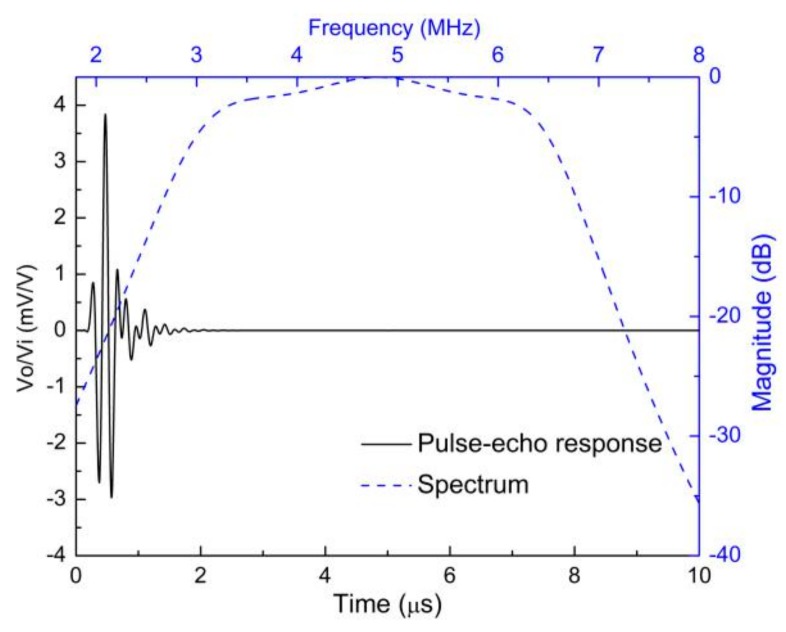
The modeled pulse–echo response and the FFT spectrum of the 5 MHz transducer.

**Figure 8:**
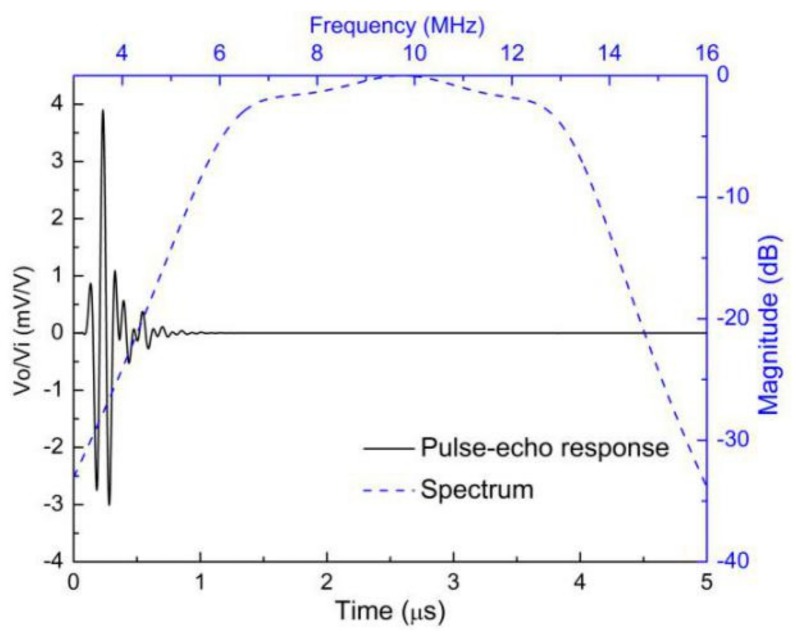
The modeled pulse–echo response and the FFT spectrum of the 10 MHz transducer.
